# Early Enteral Nutrition Could Be Associated with Improved Survival Outcome in Cardiac Arrest

**DOI:** 10.1155/2024/9372015

**Published:** 2024-06-08

**Authors:** Jingwei Duan, Jianjie Ren, Xiaodan Li, Lanfang Du, Baomin Duan, Qingbian Ma

**Affiliations:** ^1^Emergency Department, Peking University Third Hospital, Beijing, China; ^2^Emergency Department, Kaifeng Central Hospital, Kaifeng, China

## Abstract

**Background:**

Although the latest European and US guidelines recommend that early enteral nutrition (EN) be attempted in critically ill patients, there is still a lack of research on feeding strategies for patients after cardiac arrest (CA). Due to the unique pathophysiology following CA, it remains unknown whether evidence from other diseases can be applied in this condition.

**Objective:**

We aimed to explore the relationship between the timing of EN (within 48 hours or after 48 hours) and clinical outcomes and safety in CA.

**Method:**

From the MIMIC-IV (version 2.2) database, we conducted this retrospective cohort study. A 1 : 1 propensity score matching (PSM) analysis was also conducted to prevent potential interference from confounders. Moreover, adjusted proportional hazards model regression models were used to adjust for prehospital and hospitalization characteristics to verify the independence of the association between early EN initiation and patient outcomes.

**Results:**

Of the initial 1286 patients, 670 were equally assigned to the early EN or delayed EN group after PSM. Patients in the early EN group had improved survival outcomes than those in the delayed EN group within 30 days (HR = 0.779, 95% confidence interval [CI] [0.611-0.994], *p* = 0.041). Similar results were shown at 90 and 180 days. However, there was no significant difference in neurological outcome between the two groups at 30 days (51% vs. 57%, odds ratio [OR] = 0.786, 95% CI [0.580–1.066], *p* = 0.070). Patients who underwent early EN had a lower risk of ileus than patients who underwent delayed EN (4% vs. 8%, OR = 0.461, 95% CI [0.233–0.909], *p* = 0.016). Moreover, patients who underwent early EN had shorter hospital stays.

**Conclusion:**

Early EN could be associated with improved survival outcomes for patients after CA. Further studies are needed to verify it. However, at present, we might consider early EN to be a more suitable feeding strategy for CA.

## 1. Introduction

Cardiac arrest (CA) poses a serious threat to human life because of its extremely high mortality rate. Despite the latest US and European guidelines optimizing the chain of survival for CA, the survival rate at hospital discharge is still less than 10% for out-of-hospital cardiac arrest (OHCA) and less than 20% for in-hospital cardiac arrest (IHCA) in the United States [1, 2]. This leads us to wonder why patients with CA still have such poor survival outcomes. Many previous studies have focused on the process of cardiopulmonary resuscitation (CPR) and the control of vital signs after the return of spontaneous circulation (ROSC) [3, 4]. The evidence drawn from these studies continues to optimize our life-saving processes, but the high mortality rate suggests that there are still important components that are being overlooked. Most of these issues focus on postresuscitation care, such as glucose control, glucocorticoid use, and the timing of enteral nutrition (EN).

The timing of EN for critically ill patients is a topic that is both ancient and always controversial [5]. Since most patients admitted to the intensive care unit (ICU) are in a state of high metabolism and high consumption, timely administration of EN can better meet the caloric needs of critically ill patients and maintain the function of vital organs [6]. However, most of these patients are in shock, and EN seems to be a potential contraindication. Both ESPEN and ASPEN recommended that early EN (within 48 hours) can be attempted in critically ill patients, which may be associated with improved prognosis [7, 8]. However, the evidence on which this recommendation is based is largely from patients in septic shock or on mechanical ventilation [9, 10]. There is still a lack of experimental evidence on the optimal timing of EN after CA. In addition, studies related to postcardiac arrest syndrome have shown that ischemia—reperfusion injury is more common after CA. Therefore, the timing of EN is still an urgent problem. We conducted this study to evaluate the impact of early or delayed EN on clinical outcomes in patients with CA.

## 2. Materials and Methods

### 2.1. Study Design

A retrospective cohort study was conducted from a large electronic database, Medical Information Mart for Intensive Care IV (MIMIC-IV). The MIMIC-IV database contains four detailed parts (emergency department, admissions, intensive care unit, and follow-up). All the data were extracted from the latest version of the MIMIC-IV (version 2.2), which was released in January 2023 and contains information on more than four hundred thousand admissions from 2008 to 2019. Before accessing this database, CITI Data or Specimens Only Research needs to be certified (our certification is verified at: https://www.citiprogram.org/verify/?k026a8801-1124-40bb-ac72-c64d66bdab42-36003834), and the PhysioNet Credentialed Health Data Use Agreement 1.5.0, which contains the ethical statement, needs to be agreed upon [11–13].

### 2.2. Inclusion and Exclusion Criteria

This study has the following inclusion criteria: (a) all patients diagnosed with cardiac arrest (ICD-9 is 4275 and ICD-10 is I469, I468, and I469); (b) age over eighteen years; (c) any initial rhythm; (d) OHCA or IHCA.

Exclusion criteria: (a) pregnant; (b) CA caused by operation or traumas; (c) received only PN during hospitalization; (d) patients with intestinal obstruction, gastrointestinal bleeding, and other contraindications of EN before CA; (e) receiving any abdomen operation or any other examination or operation needed to pause EN during 48 hours; (f) any malignant tumor; (g) moribund patients died within 48 hours of admission; (h) incomplete information.

### 2.3. Variable Extraction

In the part of emergency department, vital signs and arterial blood gas at admission were extracted. Moreover, a free-text field that included the patient's reported reason for presenting to the emergency department was extracted from the MIMIC-IV-ED (version 2.2) [11, 14]. In the part of admissions, age, sex, previous disease, and therapy during the first two days (coronary angiography, mechanical circulation support (MCS), and therapeutic hypothermia) were extracted. Moreover, the length of ICU stay and length of hospital stay were also extracted. In the part of intensive care unit, ventilator during hospitalization, medication use during hospitalization, and the first Sequential Organ Failure Assessment (SOFA) score at the ICU were extracted. During the part of follow-up, the cumulative survival days and safety endpoints (ileus, aspiration pneumonia, and refeeding syndrome) were extracted. The above variables were extracted byfrom the ICD-9 or 10, hadm_id or stay_id, which is a unique number for every variable or patient in both the MIMIC-IV and MIMIC-IV-ED databases.

### 2.4. Endpoint Definition

We defined the primary endpoint as cumulative survival during 30 days. The safety endpoints encompassed any ileus, aspiration pneumonia, or refeeding syndrome. Moreover, the secondary endpoints were defined as cumulative survival during 90 days and 180 days, poor neurologic outcome (cerebral performance category (CPC) 3–5) [15, 16], and length of hospital and ICU stay.

Early EN was defined as receiving EN within 48 hours of admission, and delayed EN was defined as receiving EN 48 hours after admission.

### 2.5. Statistical Analysis

Continuous variables are presented as the median (interquartile range, IQR) for non-normal distribution or mean (standard deviation, SD) for normal distribution. Total number and percentage are presented for categorical variables. The Student's *t*-test is used to compare continuous variables presented as normal distribution and Mann–Whitney *U* test for non-normal distribution. *X*^2^ test or Fisher's exact test is used to compare categorical variables. The *p* value <0.05 was considered statistically significant.

We make a 1 : 1 matched propensity score-matched (PSM) analysis in order to balance possible confounders between early EN and delayed EN. The caliper of PSM was set as 0.02 for standard deviation of the logit of the propensity score. Cases with higher propensity scores will be matched first. In order to better eliminate the result bias caused by confounding factors, all listed variables in Table 1 except clinical outcome (mortality at 30, 90, and 180 days), neurologic outcome at 30 days, day of ICU stay, and hospital stay and safety endpoints (ileus, aspiration pneumonia, and refeeding syndrome) were included in the model of 1 : 1 PSM.

Kaplan–Meier and Cox-proportional hazard model were used for calculation of hazard ratio, and a confidence interval (CI) of 95% (*p* < 0.05) was considered statistically significant. Adjusted proportional hazards model (COX) regression models were used to verify the independence of association between early nutrition initiation and survival outcomes to control for prehospital characteristics (age, gender, body mass index, all included comorbidities, OHCA, bystander CPR, time from collapse to CPR, time from collapse to ROSC, and initial rhythm shockable) and hospitalization variables (vital signs, arterial blood gas, hypothermia, coronary arteriography/percutaneous coronary intervention, ventilator, mechanical circulatory support devices, vasoactive-inotropic score (VIS) and SOFA score). Moreover, subgroup analysis was also conducted to compare the difference of primary endpoint in patients with certain conditions.

All statistical analyses were performed using SPSS version 25.0 (SPSS Inc., Chicago, IL, USA) and R Programming Language version 4.2.3.

## 3. Results

**Figure 1:**
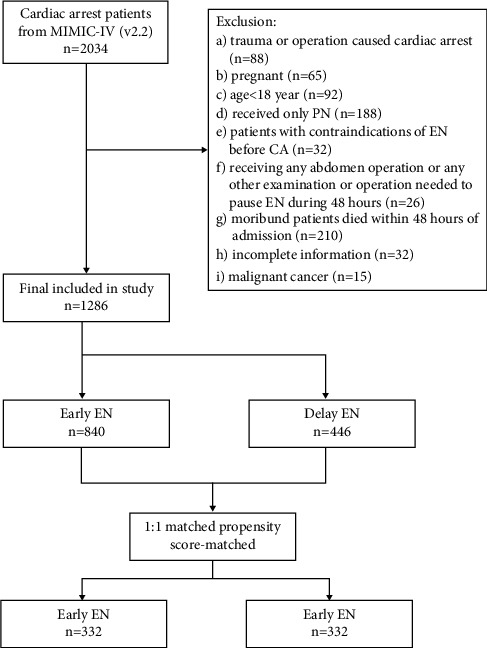
Flowchart. CA: cardiac arrest, EN: enteral nutrition, PN: parenteral nutrition.

From the initially identified 2,034 patients, a total of 1,286 patients were eligible for the study. Of these 1,286 patients, 840 patients (65%) were treated with early EN and 446 patients (34%) with delayed EN (Figure 1). After 1 : 1 matched PSM, a total of 664 patients were equally divided into early EN and delayed EN groups.

### 3.1. Baseline Characteristics

The crude baseline characteristics showed that the median age of overall patients was 66 years (IQR 55–77) and 802/1,286 (62%) patients were male. The median body mass index (BMI) was 27.5 kg/m^2^ (23.7–32.4). Most of patients were OHCA 1009/1286 (78%), and more OHCA in the early EN group than in the delayed EN group (82% vs. 72%, *p* < 0.001). However, a higher percentage of patients in the delayed EN group received bystander CPR. More patients have previous myocardial infarction in early EN group than delayed EN group (34% vs. 26%, *p*=0.002), and more previous renal dysfunction in delayed EN than early EN group (37% vs. 29%, *p*=0.002). The median of time from collapse to CPR and collapse to ROSC was 5 minutes (IQR 2–6) and 21 minutes (IQR 18–25), and there was no significant statistical difference between two groups. Patients in the delayed EN group had lower blood pressure on admission than those in the early EN group, and also had faster heart rates. More patients received ventilator in delayed EN than in early EN. Patients in the delayed EN group might be worse, because their SOFA scores were significantly higher than those in the early EN group (9 [6–12] vs. 8 [4–11], *p* < 0.001).

Regarding clinical outcomes, patients in the early EN group had significantly lower mortality at 90 and 180 days than did those in the late EN group (44% vs. 53%, *p*=0.002; 48% vs. 57%, *p*=0.001, respectively), and the percentage of patients with poor neurologic outcome (CPC 3–5) at 30 days was greater in the delayed EN group than in the early EN group (58% vs. 50%, *p*=0.001). However, the mortality at 30 days did not show significantly different between the two groups (38% vs. 41%, *p*=0.149). Regarding safety endpoints, patients in the delayed EN group had a greater occurrence of ileus than did those in the early EN group (8% vs. 4%, *p*=0.001). However, the occurrence of aspiration pneumonia and refeeding syndrome did not show significantly different between the two groups. Finally, although the total length of hospital stay was significantly longer in the delayed EN group than in the early EN group, there was no significant difference in the length of ICU stay between the two groups. The detailed crude baseline characteristics are shown in Table 1.

### 3.2. PSM Analysis

The median age of overall patients was 66 years (IQR 55–76) and 401/664 (60%) patients were male. The BMI of overall patients was 27.5 kg/m^2^ (23.8–32.1). 74% of patients were OHCA, 26% received bystander CPR, 65% received therapeutic hypothermia and only 7% had a shockable initial rhythm. The median of time for collapse to CPR and collapse to ROSC was 5 minutes (IQR 2–6) and 21 minutes (IQR 18–25), respectively. In the PSM analysis, every baseline characteristic between two groups showed no statistical difference (Table 2).

**Figure 2:**
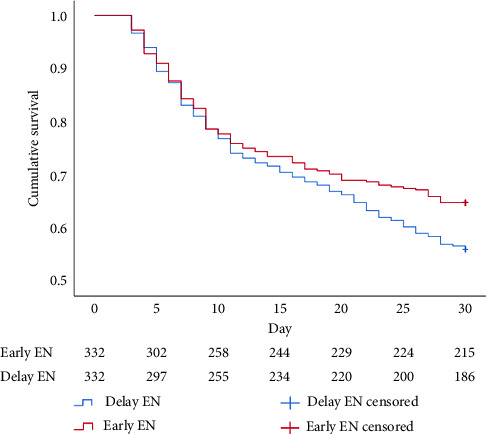
Kaplan–Meier survival curve during 30 days for PSM.

**Figure 3:**
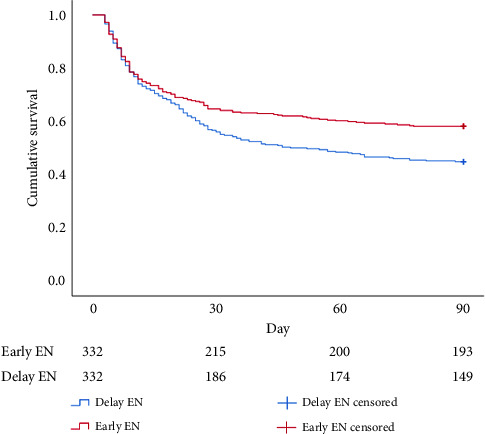
Kaplan–Meier survival curve during 90 days for PSM.

**Figure 4:**
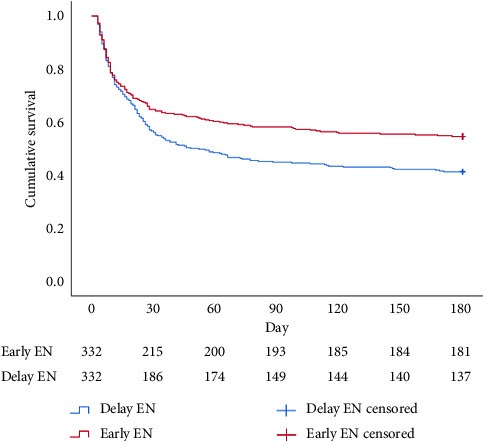
Kaplan–Meier survival curve during 180 days for PSM.

Regarding cumulative survival analysis, patients in the early EN group had better survival outcome within 30 days than patients in the delayed EN group (hazard ratio [HR] = 0.779, 95% CI [0.611–0.994], *p*=0.041) (Figure 2). Moreover, patients in the early EN group also had significantly better cumulative survival at 90 and 180 days than did those in the delayed group (HR = 0.715, 95% CI [0.574–0.892], *p*=0.003; HR = 0.719, 95% CI [0.581–0.890], *p*=0.002, respectively) (Figures 3 and 4). Moreover, the occurrence of poor neurologic outcome at 30 days was lower in the early EN group (49% vs 58%, odds ratio [OR] = 0.838, 95% CI [0.717–0.979], *p*=0.029). After adjusting for prehospital or/and hospitalization characteristics, early EN was independently associated with survival outcomes (Supplementary Table 1).

Regarding safety endpoints, the occurrence of ileus was significantly greater in the delayed EN group than in the early EN group (3% vs. 8%, OR = 0.677, 95% CI [0.548–0.838], *p*=0.008). However, other safety endpoints were not significantly different between the two groups.

Furthermore, although there was no significant difference in the length of ICU stay between the two groups, the length of hospital stay was significantly longer in the delayed EN group than in the early EN group (10 days [5–19] vs. 16 days [9–26], *p* < 0.001). The detailed results are shown in Table 3.

### 3.3. Subgroup Analysis

**Figure 5:**
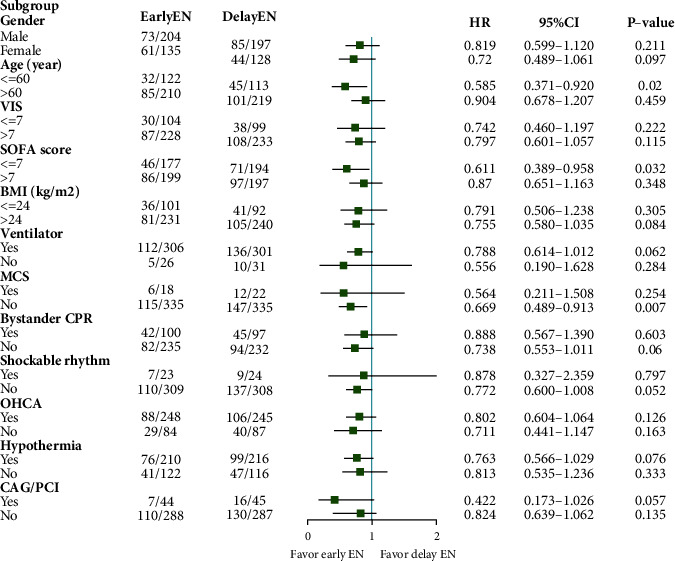
Results of subgroup analyses for primary endpoints. BMI: body mass index, CAG: coronary arteriography, CPR: cardiopulmonary resuscitation, EN: enteral nutrition, MCS: mechanical circulatory support, OHCA: out-of-hospital cardiac arrest, PCI: percutaneous coronary intervention, SOFA: sequential organ failure assessment, VIS: vasoactive-inotropic score.

Based on the PSM analysis, we performed subgroup analyses according to sex, age, VIS, SOFA score, BMI, ventilator, OHCA, MCS, hypothermia, initial shockable rhythm, bystander CPR, and CAG/PCI, respectively. We found that patients in the early EN group who were younger than 60 years (HR = 0.585, 95% CI [0.371–0.920, *p*=0.020]), without MCS (HR = 0.669, 95% CI [0.489–0.913, *p*=0.007]) or SOFA ≤ 7 (HR = 0.611, 95% CI [0.389–0.958, *p*=0.032]) had better survival outcomes than did those in the delayed EN group (Figure 5).

## 4. Discussion

According to the findings of this retrospective cohort study, early EN might be associated with improved survival outcomes after CA.

When ROSC is reached in cardiac arrest, most of the patients' enteral nutrition is initiated in the ICU. Early view states that as early EN increases the perfusion of mesenteric arteries, this affects the overall circulatory status and leads to reduced perfusion levels in vital organs such as the kidneys, heart, and brain [17]. This phenomenon is particularly pronounced in patients with shock [18]. Therefore, clinicians always consider administering enteral nutrition to patients only after their circulation levels have stabilized [19]. However, numerous recent studies have challenged this view and demonstrated that early EN may improve clinical outcomes in critically ill patients [8, 20, 21]. A previous meta-analysis of 18 randomized controlled trials (RCTs) showed that, compared with parenteral nutrition, EN does not increase the risk of mortality for critically ill patients [22]. Similarly, a systemic review showed that, compared with delayed EN (after 48 hours), early EN does not increase the risk of mortality or EN-related complications in critically ill patients [23]. Furthermore, a meta-analysis with a low level of evidence showed that early EN might reduce the risk of mortality among critically ill patients with COVID-19 [24]. Therefore, the latest European guidelines recommend that EN be safely started within 48 h after admission [25]. Although the guidelines give a recommendation, this recommendation is not supported by evidence from patients after CA.

Due to the unique pathophysiological characteristics of postcardiac arrest syndrome, we should reevaluate the use of EN in CA. During cardiac arrest, organs and tissues throughout the body remain in a state of extreme ischemia and hypoxia, despite high-quality CPR support [26]. After ROSC, organs and tissues are exposed to damage mainly from ischemia—reperfusion injury, which is caused mainly by irreversible damage caused by the activation of a large number of inflammatory mediators, resulting in the apoptosis of functional cells [27, 28]. Although the intestine tolerates ischemia and hypoxia relatively well compared to other vital organs, it is still damaged after ischemia—reperfusion. The intestine, as an immune barrier in vivo, stops bacteria in the intestinal lumen from moving [29], which could exacerbate local damage or even cause activation of systemic immune responses, thus aggravating immune inflammatory damage [30]. Therefore, early EN might maintain the integrity of the intestinal barrier by enhancing perfusion of the intestine [31]. These findings show that early EN seems reasonable for patients in CA.

Another concern regarding patients receiving hypothermia is that their metabolic levels are reduced in the hypothermic state, leading to suppressed cellular activity [32]. Consequently, current clinical practice tends to favor delayed EN for patients undergoing hypothermia treatment. However, a recent study demonstrated that, compared to delayed EN, early EN does not increase the risk of mortality or EN-related complications in patients with CA who received hypothermia [33]. This result is also consistent with our study. Patients with an initial shockable rhythm might have better clinical outcomes. Additionally, according to the PSM, only 7% of patients experienced an initial shockable rhythm. However, based on previous studies and investigations, the proportion of patients with an initial shockable rhythm was 27%–49% [34, 35]. Although the subgroup analysis suggested that the initial rhythm might not be a potential confounder for the timing of EN, the small sample size of initial shockable rhythms did not fully represent the current characteristics of CA. Therefore, studies with more balanced baseline characteristics are needed to validate the role of EN in CA patients.

Subgroup analyses show that age <60 years, SOFA score ≤7 or not receiving MCS were associated with improved survival in patients who received early EN. Both of young patients and SOFA score ≤7 indicate that the patient's condition may be less severe [36, 37]. Therefore, these patients may tolerate EN well, and in addition, the possible potential protective mechanisms of early EN may be better reflected in this population. Although survival outcomes are better in early enteral nutrition patients receiving MCS, the confidence interval for the results is too wide, so this result still needs to be considered with caution. Moreover, we noticed that the severity of disease in patients receiving different MCS may vary considerably, and this has a significant impact on the survival outcome. However, we did not perform a subgroup analysis of this because the number of patients receiving MCS was too small in this study.

In summary, this study provides evidence supporting the use of feeding strategies in cardiac arrest patients, but the underlying mechanisms still need to be further explored.

### 4.1. Limitations

Although we have made efforts to minimize the potential bias, this study still has following limitations: (a) this is a single-center retrospective cohort study, so the conclusions were needed to verify by multicenter studies; (b) due to the limitations of the data in the database, we cannot extract data on caloric intake, such as lack of assessment of protein and nonprotein kcal; (c) lack of assessment of volume and method of enteral feeding (continuous or intermittent); (d) the absence of prior randomization could have allowed subjective clinician behavior to influence patient intervention and outcomes; (e) lack of data on nutritional scores hindered our ability to assess whether a patient's nutritional status influenced the timing of EN introduction; (f) due to database limitations, we were unable to classify the cause of CA, which may have had some impact on the results; (g) lack of assessment and comparison of post-CA left ventricular ejection fraction that could reflect risk of low cardiac output syndrome. This may be a significant problem in patients after cardiac arrest.

## 5. Conclusions

Early EN might improve short-term survival outcomes, and this trend was more pronounced after extended follow-up. Moreover, early EN could decrease the occurrence of ileus but did not increase the occurrence of other EN-related complications compared with delayed EN. Therefore, administering early EN for patients after CA might be a more suitable feeding strategy for improving survival outcome. However, further randomized controlled trials are urgently needed to verify this strategy.

## Figures and Tables

**Table 1:** Crude baseline characteristics and clinical outcomes.

	All patients *N* = 1286	Early EN *N* = 840	Delayed EN *N* = 446	*p* value
Age (year), median (IQR)	66 (55–77)	65 (54–76)	66 (56–78)	0.216
Male, *n* (%)	802 (62)	526 (63)	276 (62)	0.421
Smoking, *n* (%)	355 (26)	239 (28)	116 (26)	0.193
BMI (kg/m^2^), median (IQR)	27.5 (23.7–32.4)	27.5 (24.0–32.0)	27.4 (23.3–32.7)	0.913
*Previous diseases*				
Myocardial infarction, *n* (%)	404 (31)	287 (34)	117 (26)	0.002
Heart failure, *n* (%)	580 (45)	385 (46)	195 (44)	0.253
Diabetes mellitus, *n* (%)	488 (38)	307 (37)	181 (41)	0.087
Renal insufficiency, *n* (%)	404 (31)	240 (29)	164 (37)	0.002
OHCA, *n* (%)	1009 (78)	687 (82)	322 (72)	<0.001
Initial shockable rhythm, *n* (%)	93 (7)	58 (7)	35 (8)	0.303
Bystander CPR, *n* (%)	333 (26)	190 (23)	143 (32)	<0.001
Time from collapse to CPR, median (IQR)	5 (2–6)	5 (2–6)	4 (3–7)	0.614
Time from collapse to ROSC, median (IQR)	21 (18–25)	21 (18–25)	21 (18–25)	0.975
*Vital sign at admission*				
SBP, mmHg, median (IQR)	85 (76–96)	87 (77–97)	83 (74–93)	<0.001
DBP, mmHg, median (IQR)	44 (37–51)	44 (38–52)	42 (36–50)	<0.001
MAP, mmHg, median (IQR)	57 (49–63)	57 (50–64)	55 (47–62)	<0.001
Heart rate max first day, median (IQR)	102 (88–120)	101 (87–119)	103 (90–120)	0.015
Lactate, mmol/L, median (IQR)	3.2 (1.6–6.6)	3.3 (1.7–6.9)	3.1 (1.5–6.1)	0.124
pH, median (IQR)	7.26 (7.12–7.35)	7.25 (7.12–7.34)	7.26 (7.12–7.36)	0.433
Therapeutic hypothermia, *n* (%)	841 (65)	556 (66)	285 (64)	0.223
^#^CAG/PCI, *n* (%)	183 (14)	124 (15)	59 (13)	0.254
Ventilator, *n* (%)	1118 (89)	706 (84)	412 (92)	<0.001
^$^Mechanical circulatory support device, *n* (%)	78 (6)	47 (6)	31 (7)	0.198
*Medications*				
Muscle relaxant, *n* (%)	189 (15)	116 (14)	73 (16)	0.125
Epinephrine, *n* (%)	487 (38)	320 (38)	167 (37)	0.434
Norepinephrine, *n* (%)	761 (59)	496 (59)	265 (59)	0.473
Dopamine, *n* (%)	213 (17)	140 (17)	73 (16)	0.479
Vasopressin, *n* (%)	266 (21)	152 (18)	114 (26)	0.001
Dobutamine, *n* (%)	62 (5)	39 (5)	23 (5)	0.388
^*∗*^Max VIS, median (IQR)	14 (3–25)	13 (0–24)	15 (4–27)	0.045
SOFA score at admission, median (IQR)	8 (5–12)	8 (4–11)	9 (6–12)	<0.001
ICU stays (day)	3 (2–7)	3 (2–7)	3 (2–7)	0.744
Hospital stays (day)	11 (6–20)	9 (5–16)	16 (9–26)	<0.001
Mortality at 30 days	507 (39)	322 (38)	185 (41)	0.149
Mortality at 90 days	609 (47)	372 (44)	237 (53)	0.002
Mortality at 180 days	654 (51)	400 (48)	254 (57)	0.001
CPC 3–5 at 30 days	660 (51)	403 (50)	257 (58)	0.001
*Safety endpoints during 30 days*				
Ileus	69 (5)	32 (4)	37 (8)	0.001
Aspiration pneumonia	136 (11)	81 (10)	55 (12)	0.082
Refeeding syndrome	53 (6)	34 (4)	19 (4)	0.480

BMI: body mass index, CAG: coronary arteriography, CPC: cerebral performance category, CPR: cardiopulmonary resuscitation, DBP: diastolic blood pressure, EN: enteral nutrition, ICU: intensive care unit, MAP: mean arterial pressure, OHCA: out-of-hospital cardiac arrest, PCI: percutaneous coronary intervention, ROSC: return of spontaneous circulatory, SBP: systolic blood pressure, SOFA: sequential organ failure assessment, VIS: vasoactive-inotropic score. ^#^patient received coronary arteriography or percutaneous coronary intervention within the 48 hours of admission. ^$^Mechanical circulatory support device included extracorporeal membrane oxygenation, intraaortic ballon pump, impella, tandemheart. ^*∗*^Max VIS is the maximum within 48 hours of admission.

**Table 2:** Baseline characteristics for PSM.

	All patients *N* = 664	Early EN *N* = 332	Delayed EN *N* = 332	*p* value
Age (year), median (IQR)	66 (55–76)	66 (55–76)	66 (56–78)	0.469
Male, *n* (%)	401 (60)	204 (61)	197 (59)	0.634
Smoking, *n* (%)	188 (28)	98 (30)	90 (27)	0.547
BMI (kg/m^2^), median (IQR)	27.6 (23.8–32.1)	27.3 (23.0–32.9)	27.6 (24.1–31.8)	0.909
*Previous diseases*				
Myocardial infarction, *n* (%)	178 (27)	88 (27)	90 (27)	0.930
Heart failure, *n* (%)	281 (42)	136 (41)	145 (44)	0.530
Diabetes mellitus, *n* (%)	264 (40)	128 (39)	136 (41)	0.579
Renal insufficiency, *n* (%)	243 (37)	118 (36)	125 (38)	0.629
OHCA, *n* (%)	493 (74)	248 (75)	245 (74)	0.859
Initial shockable rhythm, *n* (%)	47 (7)	23 (7)	24 (7)	1.000
Bystander CPR, *n* (%)	197 (30)	97 (29)	100 (30)	0.865
Time from collapse to CPR, median (IQR)	5 (3–7)	4 (3–7)	4 (2–6)	0.868
Time from collapse to ROSC, median (IQR)	22 (18–26)	21 (17–25)	21 (18–25)	0.922
*Vital sign at admission*				
SBP, mmHg, median (IQR)	84 (76–93)	84 (76–94)	83 (75–93)	0.335
DBP, mmHg, median (IQR)	43 (36–50)	43 (36–51)	42 (36–50)	0.591
MAP, mmHg, median (IQR)	56 (48–62)	57 (50–64)	55 (47–62)	0.889
Heart rate max first day, median (IQR)	103 (89–121)	104 (89–120)	103 (90–120)	0.894
Lactate, mmol/L, median (IQR)	3.1 (1.6–6.1)	3.0 (1.6–6.1)	3.1 (1.6–6.1)	0.936
pH, median (IQR)	7.25 (7.12–7.35)	7.26 (7.14–7.35)	7.26 (7.11–7.35)	0.879
Therapeutic hypothermia, *n* (%)	426 (64)	210 (63)	216 (65)	0.686
CAG/PCI, *n* (%)	89 (13)	44 (13)	45 (14)	1.000
Ventilator, *n* (%)	607 (91)	306 (92)	301 (91)	0.580
^$^Mechanical circulatory support device, *n* (%)	40 (6)	18 (5)	22 (7)	0.625
*Medication*				
Muscle relaxant, *n* (%)	112 (17)	57 (17)	55 (17)	0.918
Epinephrine, *n* (%)	248 (37)	123 (37)	125 (38)	0.936
Norepinephrine, *n* (%)	401 (60)	203 (61)	198 (60)	0.751
Dopamine, *n* (%)	122 (18)	67 (20)	55 (17)	0.270
Vasopressin, *n* (%)	146 (00)	67 (20)	79 (24)	0.303
Dobutamine, *n* (%)	35 (5)	16 (5)	19 (6)	0.729
^*∗*^Max VIS, median (IQR)	15 (4–25)	15 (4–25)	15 (4–26)	0.714
SOFA score at admission, median (IQR)	9 (5–12)	9 (5–12)	9 (5–12)	0.974

BMI: body mass index, CAG: coronary arteriography, CPR: cardiopulmonary resuscitation, DBP: diastolic blood pressure, EN: enteral nutrition, MAP: mean arterial pressure, OHCA: out-of-hospital cardiac arrest, PCI: percutaneous coronary intervention, ROSC: return of spontaneous circulatory, SBP: systolic blood pressure, SOFA: sequential organ failure assessment, VIS: vasoactive-inotropic score. ^#^patient received coronary arteriography or percutaneous coronary intervention within the 48 hours of admission. ^$^Mechanical circulatory support device included extracorporeal membrane oxygenation, intraaortic ballon pump, impella, tandemheart. ^*∗*^Max VIS is the maximum within 48 hours of admission.

**Table 3:** Clinical outcome for PSM.

	Early EN *N* = 332	Delayed EN *N* = 332	Hazard ratio	95% CI	*p* value
Cumulative mortality during 30 days			0.779	0.611–0.994	0.041
Cumulative mortality during 90 days			0.715	0.574–0.892	0.003
Cumulative mortality during 180 days			0.719	0.581–0.890	0.002

	Odds ratio				

CPC 3–5 during 30 days	162 (49)	191 (58)	0.838	0.717–0.979	0.029
*Safety endpoints at 30 days*					
Aspiration pneumonia	29 (9)	35 (11)	0.905	0.714–1.363	0.511
Ileus	11 (3)	28 (8)	0.677	0.548–0.838	0.008
Refeeding syndrome	17 (5)	14 (4)	1.112	0.749–1.652	0.741
ICU stays (day), median (IQR)	3 (2–7)	3 (2–7)			0.728
Hospital stays (day), median (IQR)	10 (5–19)	16 (9–25)			<0.001

CPC: cerebral performance category, EN: enteral nutrition, ICU: intensive care unit.

## Data Availability

MIMIC-IV is an electronic database of credentialed access (URL: https://physionet.org/content/mimiciv/2.2/). Before you can get access, you have to receive training of CITI Data or Specimens Only Research (URL: https://physionet.org/content/mimiciv/view-required-training/2.2/#1) and pass the test of COLLABORATIVE INSTITUTIONAL TRAINING INITIATIVE (CITI PROGRAM) COMPLETION REPORT - PART 1 OF 2 (URL: https://about.citiprogram.org/). After you have obtained a certificate (it has a unique number to recognize), you will have the right to apply for and, upon passing the application, to use the MIMIC-IV database.

## Supplementary Materials

Supplementary Materials Results of adjusted proportional hazards model (COX) regression models. Adjusting confounding factors included prehospital and hospitalization characteristics for 30-day, 90-day, and 180-day cumulative mortality.
