# Supplementary material for: Early Enteral Nutrition Could Be Associated with Improved Survival Outcome in Cardiac Arrest
Source: Emerg Med Int. 2024 Jun 8;2024:9372015. doi: 10.1155/2024/9372015 (PMC11221999; doi:10.1155/2024/9372015)
Supplement: Supplementary Materials — Results of adjusted proportional hazards model (COX) regression models. Adjusting confounding factors included prehospital and hospitalization characteristics for 30-day, 90-day, and 180-day cumulative mortality. [file 9372015.f1.docx]

**Supplementary Table 1.** Results of Adjusted proportional hazards model (COX) regression models

|  | **HR** | **95% CI** | **P-value** |
| --- | --- | --- | --- |
| **Adjusted proportional hazards model regression models for 30-day cumulative mortality** |  |  |  |
| EN plus prehospital characteristics^*^ | 0.787 | 0.617-0.995 | 0.048 |
| EN plus hospitalization characteristics^#^ | 0.762 | 0.597-0.974 | 0.030 |
| EN plus prehospital and hospitalization characteristics | 0.779 | 0.610-0.995 | 0.046 |
| **Adjusted proportional hazards model regression models for 90-day cumulative mortality** |  |  |  |
| EN plus prehospital characteristics^*^ | 0.721 | 0.578-0.899 | 0.004 |
| EN plus hospitalization characteristics^#^ | 0.696 | 0.557-0.869 | 0.001 |
| EN plus prehospital and hospitalization characteristics | 0.706 | 0.565-0.881 | 0.002 |
| **Adjusted proportional hazards model regression models for 180-day cumulative mortality** |  |  |  |
| EN plus prehospital characteristics^*^ | 0.724 | 0.585-0.895 | 0.003 |
| EN plus hospitalization characteristics^#^ | 0.701 | 0.566-0.869 | 0.001 |
| EN plus prehospital and hospitalization characteristics | 0.707 | 0.571-0.876 | 0.002 |

*Prehospital characteristics include gender, age, body mass index, all comorbidities, out-of-hospital cardiac arrest, bystander cardiopulmonary resuscitation, time from collapse to cardiopulmonary resuscitation, time from collapse to return of spontaneous circulation, and initial rhythm shockable.

# Hospitalization characteristics include vital signs, blood gas, coronary arteriography/percutaneous coronary intervention, hypothermia, ventilator, mechanical circulatory support devices, Sequential Organ Failure Assessment score and vasoactive-inotropic score.
